# The unique expression profile of *FAM19A1* in the mouse brain and its association with hyperactivity, long-term memory and fear acquisition

**DOI:** 10.1038/s41598-020-60266-1

**Published:** 2020-03-02

**Authors:** Hyo Jeong Yong, Nui Ha, Eun Bee Cho, Seongsik Yun, Hyun Kim, Jong-Ik Hwang, Jae Young Seong

**Affiliations:** 1 The GPCR laboratory, Graduate School of Biomedical Science, Korea University College of Medicine, Seoul, 02841 Republic of Korea; 2 Neuracle Science Co. Ltd., Seoul, 02841 Republic of Korea; 3 Department of Anatomy, Korea University College of Medicine, Seoul, 02841 Republic of Korea

**Keywords:** Neuronal development, Developmental disorders

## Abstract

Neurodevelopment and mature brain function are spatiotemporally regulated by various cytokines and chemokines. The chemokine-like neuropeptide FAM19A1 is a member of family with sequence similarity 19 (FAM19), which is predominantly expressed in the brain. Its highly conserved amino acid sequence among vertebrates suggests that FAM19A1 may play important physiological roles in neurodevelopment and brain function. Here we used a *LacZ* reporter gene system to map the expression pattern of the *FAM19A1* gene in the mouse brain. The *FAM19A1* expression was observed in several brain regions starting during embryonic brain development. As the brain matured, the *FAM19A1* expression was detected in the pyramidal cells of cortical layers 2/3 and 5 and in several limbic areas, including the hippocampus and the amygdala. FAM19A1-deficient mice were used to evaluate the physiological contribution of FAM19A1 to various brain functions. In behavior analysis, FAM19A1-deficient mice exhibited several abnormal behaviors, including hyperactive locomotor behavior, long-term memory deficits and fear acquisition failure. These findings provide insight into the potential contributions of FAM19A1 to neurodevelopment and mature brain function.

## Introduction

Developmental and physiological processes in the central nervous system (CNS) are tightly regulated by a series of orchestrated gene expressions to promote the formation of dynamic neural circuitries and to execute diverse brain functions^1,2^. These gene expressions are heavily influenced by numerous extrinsic factors, including secretory signaling molecules, extracellular matrix proteins, and membrane-bound signaling proteins. In particular, several types of secretory proteins are produced by brain cells and play crucial roles in biological processes in the CNS, including neurogenesis and synaptic plasticity^3–5^.

Cytokines and chemokines are secretory proteins that mediate a diverse range of functions in the CNS^6^. In neural induction process, cytokines are known to act as regulators in the self-renewal of neural stem cells (NSCs) and progenitor differentiation^7,8^. Moreover, chemokines, a subclass of cytokines, are involved in neural development. For instance, C-X-C motif chemokine 12 (CXCL12) regulates neural migration and axon pathfinding via the C-X-C chemokine receptor type 4 (CXCR4) signaling pathway^9,10^. Furthermore, in the adult nervous system, cytokines such as leukemia inhibitory factor (LIF) control neurotransmitter and neuropeptide profiles^11,12^, whereas interleukin-1 beta (IL-1β) modulates the activity of local neural networks via reconstructing synaptic plasticity and intercellular communication^13,14^. Taken together, these findings indicate that the normal development and physiological functions within the CNS depend on the spatiotemporal regulation of several cytokines and chemokines. Given emerging evidence that abnormal cytokine profiles are associated with neurodevelopmental disorders such as autism spectrum disorder (ASD) and attention deficit hyperactive disorder (ADHD), it is important to understand the biological functions of these small secretory proteins in the CNS^15,16^.

Family with sequence similarity 19 (FAM19) is a chemokine-like peptide family composed of five highly homologous genes, *FAM19A1* to *FAM19A5*. These genes are well conserved across species with specific expression patterns in the CNS^17^. Recent studies have shown that FAM19A2 may function as a neurotrophic factor with important roles in social novelty preference, anxiety response and neural survival^18–20^. FAM19A4 modulates mechanical and chemical-induced hypersensitivity via the regulation of neuronal excitability^21,22^. In *FAM19A3* knock-out mice, ASD-like phenotypes are observed, including increased anxiety and repetitive behaviors^23^. Furthermore, a recent study reports that FAM19A1 may regulate the proliferation and differentiation of NSCs, which implies a possible important role of FAM19A1 in brain development and neurogenesis^24^.

In this study, we generated *FAM19A1 LacZ* knock-in (KI) mice to investigate the potential roles of FAM19A1 in the brain. We demonstrated that *FAM19A1* is expressed in early stages of brain developmental with restricted expression patterns. We further demonstrated that this expression expands throughout the cortex in a cortical layer-dependent manner as the brain matures. Moreover, in the mature brain, *FAM19A1* is expressed in various regions of the limbic system. We also conducted behavioral analyses, showing that FAM19A1-deficient mice exhibit locomotor hyperactivity and deficits in long-term memory and fear acquisition. These findings suggest that FAM19A1 may be involved in both neurodevelopment and important brain functions.

## Results

### Generation of *LacZ*-expressing *FAM19A1* knock-out mice

**Figure 1:**
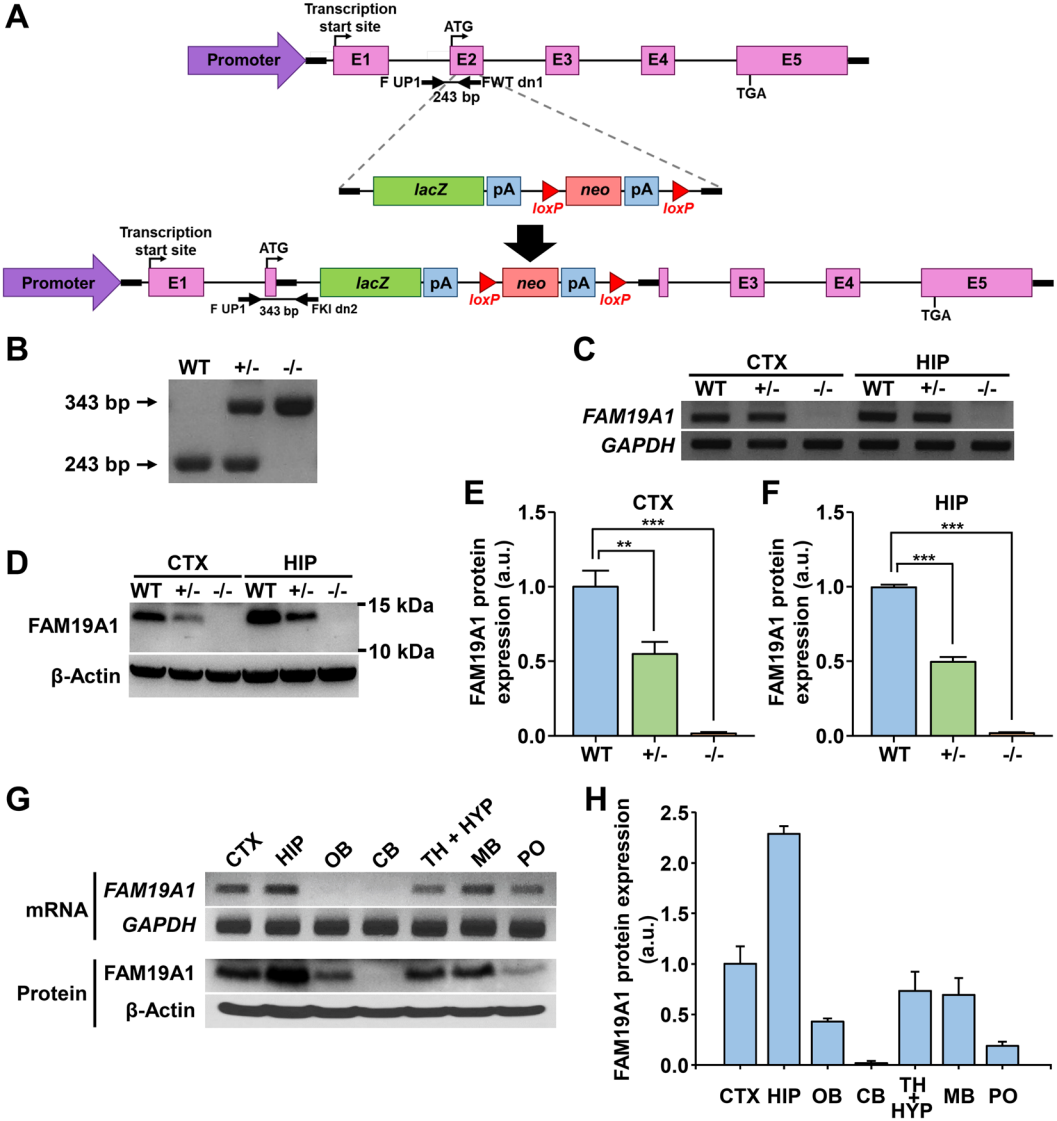
Gene construct and validation of *FAM19A1 LacZ* knock-in (KI) mice. (**A**) Schematic diagram of the *FAM19A1 LacZ* KI mouse gene construct. F UP1 is the forward primer and FWT dn1 and FKI dn2 are the reverse primers for the genotyping. (**B**) Genotypes of *FAM19A1 LacZ* KI mice were determined by genomic DNA PCR using primers targeting the inserted *LacZ* gene. (**C**) Detection of *FAM19A1* mRNA in the mouse CTX and HIP tissues by RT-PCR using primers targeting exon 2 and exon 5 of *FAM19A1*. (**D**) Detection of endogenous FAM19A1 protein in the mouse CTX and HIP tissue lysates using FAM19A1 specific antibody. Exposure time during development with ECL solution; 30 min for FAM19A1 and 1 min for β-actin. (**E**,**F**) FAM19A1 protein expression level in +/− (n = 3) and −/− (n = 3) against WT (n = 3) from western blot analysis. (**G**) Detection of *FAM19A1* mRNA and FAM19A1 protein in various WT mouse brain regions by RT-PCR and western blotting. Exposure time during development with ECL solution; 30 min for FAM19A1 and 1 min for β-actin. (**H**) FAM19A1 protein expression level in various brain regions relative to CTX of WT mice (n = 3). E1, exon 1; E2, exon 2; E3, exon 3; E4, exon 4; E5, exon 5; *lacZ*, *lacZ* gene; *neo*, amino 3′-glycosyl phosphotransferase gene; pA, poly-A tail; WT, wild type mouse; +/−, heterozygous *FAM19A1 LacZ* KI; −/−, homozygous *FAM19A1 LacZ* KI; CTX, cortex; HIP, hippocampus; OB, olfactory bulb; CB, cerebellum; TH, thalamus; HYP, hypothalamus; MB, midbrain; PO, pons; a.u., arbitrary unit. Data are presented as means ± standard errors of the means (SEM). **p < 0.01, ***p < 0.001 versus WT by one-way analysis of variance (ANOVA) with Bonferroni *post hoc* tests. Full-length gels and blots are presented in Supplementary Fig. S1 for panels B, C and D and S3 for panel G.

*FAM19A1 LacZ* KI mice were generated to investigate the role of FAM19A1 in the brain. The *LacZ* gene encoding β-galactosidase was inserted immediately after the translational start codon in exon 2 of the *FAM19A1* gene to ensure β-galactosidase production when and where *FAM19A1*is expressed (Fig. 1A). The *FAM19A1 LacZ* KI mice used in this experiment were validated by genomic DNA PCR using primers specifically targeting the inserted *LacZ* gene sequence (Fig. 1B). Given that the inserted *LacZ* gene sequence has its own stop codon as well as a poly-A tail, the final product of this gene construct is the intact β-galactosidase without any parts of FAM19A1. Disruption of the *FAM19A1* gene in *FAM19A1 LacZ* KI mice was confirmed by RT-PCR (Fig. 1C).

To validate FAM19A1 protein expression, tissue extracts of the cortex and hippocampus were analyzed by western blotting with in-house made FAM19A1-specific antibody (Fig. 1D–F and Supplementary Fig. S2). FAM19A1 was determined in wild-type (WT) mice, whereas its level was substantially decreased in heterozygous mice and was not detected in homozygous mice. In addition, it was confirmed that FAM19A1 protein was abundant where its mRNA expression was high (Fig. 1G,H).

### *FAM19A1* gene expression in the developing mouse brain

To investigate the possible involvement of FAM19A1 during neurodevelopment, the expression patterns of *FAM19A1* in embryonic and postnatal mouse brains were visualized. Because the *FAM19A1 LacZ* KI mouse was designed to express β-galactosidase instead of FAM19A1, the expression pattern of *FAM19A1* could be traced by enzymatic activity of β-galactosidase via X-gal staining. Heterozygous mice were used to analyze the expression patterns, which may avoid a potential abnormality of mice due to the complete ablation of the FAM19A1 protein.

**Figure 2:**
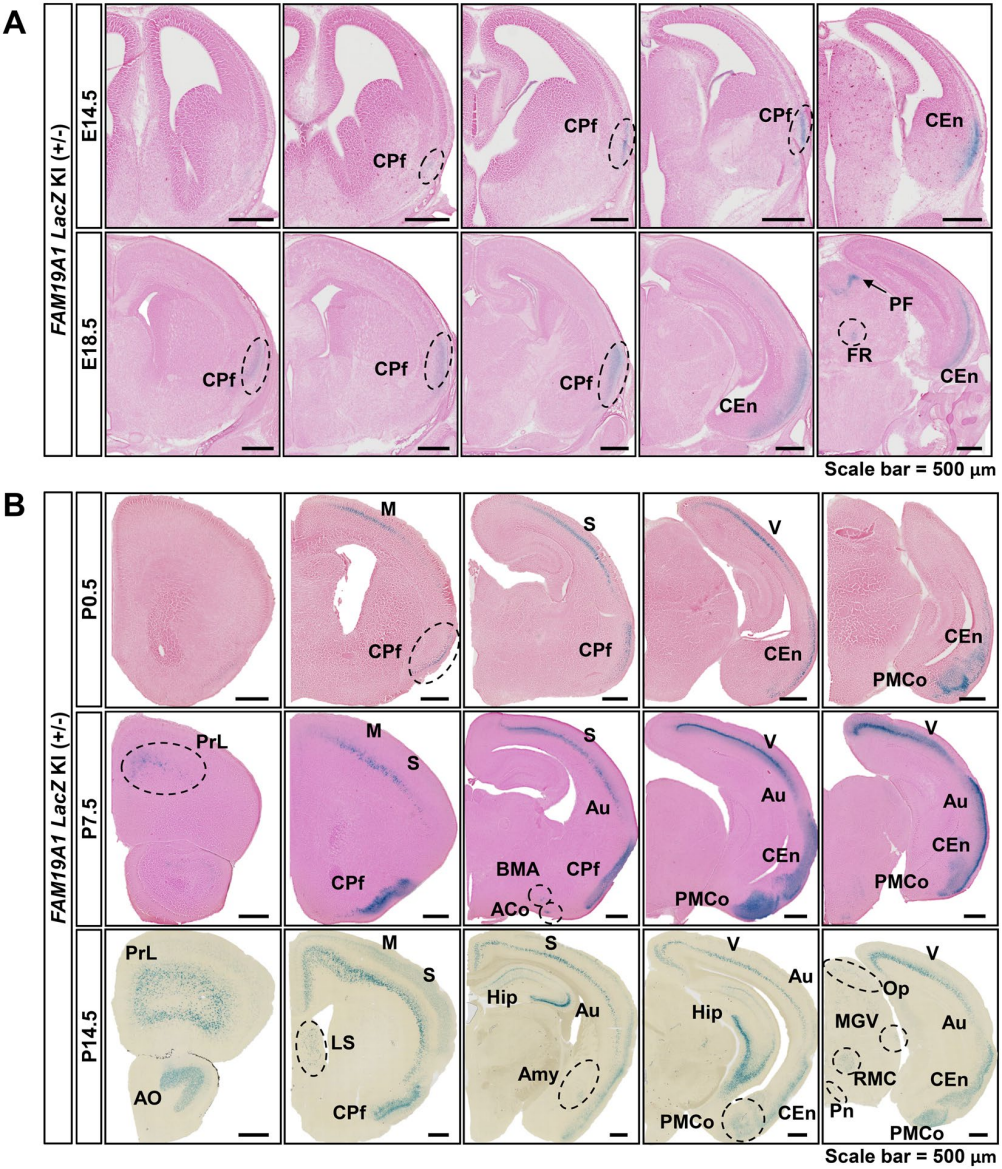
X-gal staining of embryonic and postnatal *FAM19A1 LacZ* knock-in (KI) (+/−) mouse brains. (**A**) Detection of X-gal signals in the coronal brain sections at E14.5 and E18.5. (**B**) X-gal staining of postnatal mouse brains. ACo, anterior cortical amygdaloid nucleus; Amy, amygdala; AO, anterior olfactory nucleus; Au, auditory cortex; BMA; basomedial amygdaloid nucleus, anterior part; CEn, entorhinal cortex; CPf, piriform cortex; FR, fasciculus retroflexus; Hip, hippocampus; LS, lateral septal nucleus; M, motor cortex; MGV, medial geniculate nucleus, ventral part; Op, optic nerve layer of the superior colliculus; PF, pontine flexure; PMCo, posteromedial cortical amygdaloid nucleus; Pn, pontine nuclei; PrL, prelimbic cortex; RMC, red nucleus, magnocellular part; S, somatosensory cortex; V, visual cortex. Scale bars represent 500 μm in panels A and B.

There was no sign of *FAM19A1* expression through embryonic day 12.5 (E12.5; Supplementary Fig. S4A). Starting from embryonic day 14.5 (E14.5), β-galactosidase activity was observed in the piriform cortex (CPf) and the entorhinal cortex (CEn) (Fig. 2A and Supplementary Fig. S4B). Neocortical expression of β-galactosidase was seen after birth and gradually expanded to other regions as postnatal age increased (Supplementary Fig. S4C). By postnatal day 14.5 (P14.5), neocortical β-galactosidase expression was found in a cortical layer-specific manner (Fig. 2B). In addition, X-gal staining signals were detected in the limbic areas, including the posteromedial cortical amygdaloid nucleus (PMCo), the hippocampus and the amygdala (Fig. 2B). These results provide preliminary evidence that FAM19A1 is likely expressed in differentiated neuronal cells during neurodevelopment, as *FAM19A1* expression patterns were specifically confined to the cortical layers and regions of the limbic system. In addition, *FAM19A1* was not detected in stem-cell rich regions, such as the ventricular zone or the subventricular zone, suggesting that FAM19A1 may not be involved in the proliferation of NSCs.

### *FAM19A1*gene expression in the adult mouse brain

Given the recent evidence that cytokines are important mediators in normal neural activities^25,26^, it was hypothesized that FAM19A1 may play a critical role in various neural circuits. To understand the putative contribution of FAM19A1 in mature brain functions, the expression pattern of *FAM19A1* was mapped in adult *FAM19A1 LacZ* KI heterozygous mice.

**Figure 3:**
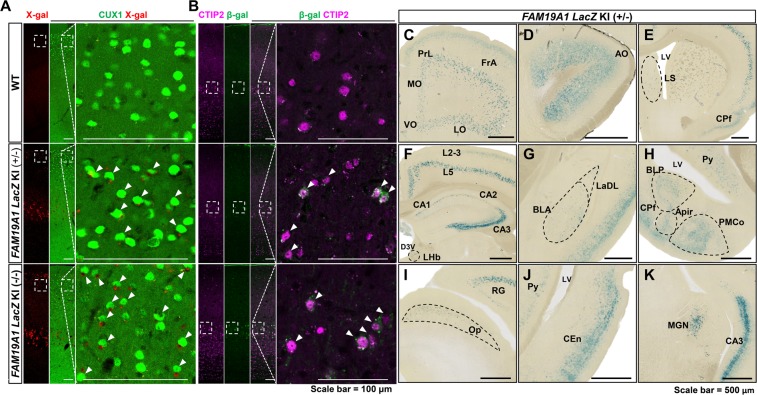
Expression pattern of *FAM19A1* in the adult mouse brain. (**A**) X-gal precipitates (red) were detected in the subset of cortical L2-3 CUX1-positive neurons (green) in *FAM19A1 LacZ* KI mice. (**B**) β-galactosidase (green) were identified in the subset of cortical L5 CTIP2-positive neurons (magenta) in *FAM19A1 LacZ* KI mice. Arrowheads indicate X-gal or β-galactosidase expressing cortical marker cells. (**C**–**K**) X-gal staining in the coronal brain sections of a *FAM19A1 LacZ* KI (+/−) mouse. AO, anterior olfactory nucleus; Apir, amygdalopiriform transition area; BLA, basomedial amygdaloid nucleus; BLP, basolateral amygdaloid nucleus, posterior part; CEn, entorhinal cortex; CPf, piriform cortex; D3V, dorsal 3rd ventricle; FrA, frontal association cortex; L2-3, cortical layer 2-3; L5, cortical layer 5; CA1, 2 and 3, field of CA1, CA2 and CA3 regions of the hippocampus; LaDL, lateral amygdaloid nucleus; LHb, lateral habenular nucleus; LO, lateral orbital cortex; LS, lateral septal nucleus; LV, lateral ventricle; MGN, medial geniculate nucleus; MO, medial orbital cortex; Op, optic nerve layer of the superior colliculus; PMCo, posteromedial cortical amygdaloid nucleus; PrL, prelimbic cortex; Py, pyramidal cell layer of the hippocampus; RG, retrosplenial granular cortex; VO, ventral orbital cortex. Scale bars represent 100 μm in panel A and 500 μm in panels (**C**–**K**).

In the adult mouse brain, X-gal staining indicated that *FAM19A1* was expressed in all cortical areas (Supplementary Fig. S4C). Immunohistochemistry with X-gal staining showed that X-gal precipitates and β-galactosidase were co-localized with CUX1, a pyramidal neuronal marker for cortical layers 2–3 (L2-3)^27^ and CTIP2, a pyramidal neuronal marker for cortical layer 5b (L5b)^27^, respectively. This indicates that* FAM19A1*is expressed mainly in pyramidal neurons in a layer-specific manner (Fig. 3A,B,F). X-gal signals in the corticospinal tract, including the internal capsule (ic), the cerebral peduncle (cp) and the pyramidal tract (py) may further support the presence of FAM19A1 in the pyramidal neurons of primary motor cortical L5b (Supplementary Fig. S5H,I).

The presence of FAM19A1 in specific sensory circuits was also investigated. In the olfactory neural circuitry, β-galactosidase and *FAM19A1* mRNA expressions were hardly observed in the olfactory bulbs (Fig. 1G and Supplementary Fig. S5A), but FAM19A1 proteins were detected by western blotting (Fig. 1G). Detected FAM19A1 proteins might be released from neurons of other olfaction-related brain regions including the anterior olfactory nucleus (AO), the CPf, and the cortical amygdala that exhibited positive X-gal signals (Fig. 3D,E,H). For the visual neural circuit, there was no β-galactosidase expression in the optic chiasm or the lateral geniculate nucleus (LGN) of the visual neural circuit, but the optic layer of the superior colliculus (Op) and the visual cortex both exhibited β-galactosidase expression (Fig. 3I and Supplementary Fig. S4C), indicating that FAM19A1 may be involved in superior colliculus-dependent visual information processing and ocular motor control. The β-galactosidase expression was also observed in some regions associated with the auditory neural circuit, including the medial geniculate nucleus (MGN), the dorsal cochlear nucleus (DC) and the auditory cortex (Fig. 3K and Supplementary Fig. S5F).

There was notable expression of *FAM19A1* in the limbic regions, including the hippocampus and the amygdala. In the hippocampus, β-galactosidase was expressed in the CA regions but not in the dentate gyrus (DG) (Fig. 3F). Previous report claimed that FAM19A1 protein was detected in both CAs and DG of the hippocampus using immunohistochemistry^24^. Open-source single cell RNA sequencing database supports our expression pattern showing lack of *FAM19A1* expression in the DG^28^. However, FAM19A1 as being a secretory protein, there is a possibility that FAM19A1 protein might be detected in non-*FAM19A1* gene expressing brain regions. With the β-galactosidase expression in the CEn, hippocampal *FAM19A1* expression suggests its possible participation in the hippocampal trisynaptic circuit (Fig. 3F,J). Among the amygdaloidal nuclei, β-galactosidase was expressed exclusively in the basolateral nuclei, including the lateral amygdaloid nucleus (LaDL) and the basomedial amygdaloid nucleus (BLA) (Fig. 3G). In addition, *FAM19A1* expression was detected in the PMCo and the amygdalopiriform transition area (Apir) (Fig. 3H), which have direct connections with the BLA, the CEn and the CPf ^29^.

The β-galactosidase was expressed in some hypothalamic nuclei, including the medial preoptic nucleus (MPOM), the lateral preoptic area (LPO) and the ventromedial hypothalamic nucleus (VMH) (Supplementary Fig. S5B,C). As a part of the limbic system, the hypothalamus acts as a mediator between the CNS and the endocrine system^30^. FAM19A1 may therefore contribute to endocrine homeostasis. The lateral septal nucleus (LS), another brain region extensively connected to the limbic areas also had the β-galactosidase expression (Fig. 3E)^31^.

*In situ* hybridization using the adult wild type rat brain showed that *FAM19A1* mRNAs were detected in the upper and lower cortical layers, the CA regions of the hippocampus and the basolateral nuclei of the amygdala (Supplementary Fig. S6A). These *FAM19A1* mRNA expression patterns coincided with the expression pattern of β-galactosidase in the *FAM19A1 LacZ* KI mouse brain, providing credibility to *FAM19A1* expression mapping using *FAM19A1 LacZ* KI mice (Fig. 3D,F,G). Moreover, our *FAM19A1* expression pattern highly agrees with open-source single cell-based RNA-sequencing database for WT mouse brain^28^. Taken together, these data indicate that *FAM19A1* is mainly expressed in neurons, particularly in pyramidal neurons, and is likely to be involved in motor behavior, sensory information processing and limbic system-related brain functions.

### Morphological differences between WT and *FAM19A1* −/− mouse brains

Early deficiency of FAM19A1 may cause developmental abnormalities in the brain. Morphological differences in homozygous *FAM19A1 LacZ* KI (*FAM19A1* −/−), heterozygous *FAM19A1 LacZ* KI (*FAM19A1* +/−) and WT mice were investigated.

**Figure 4:**
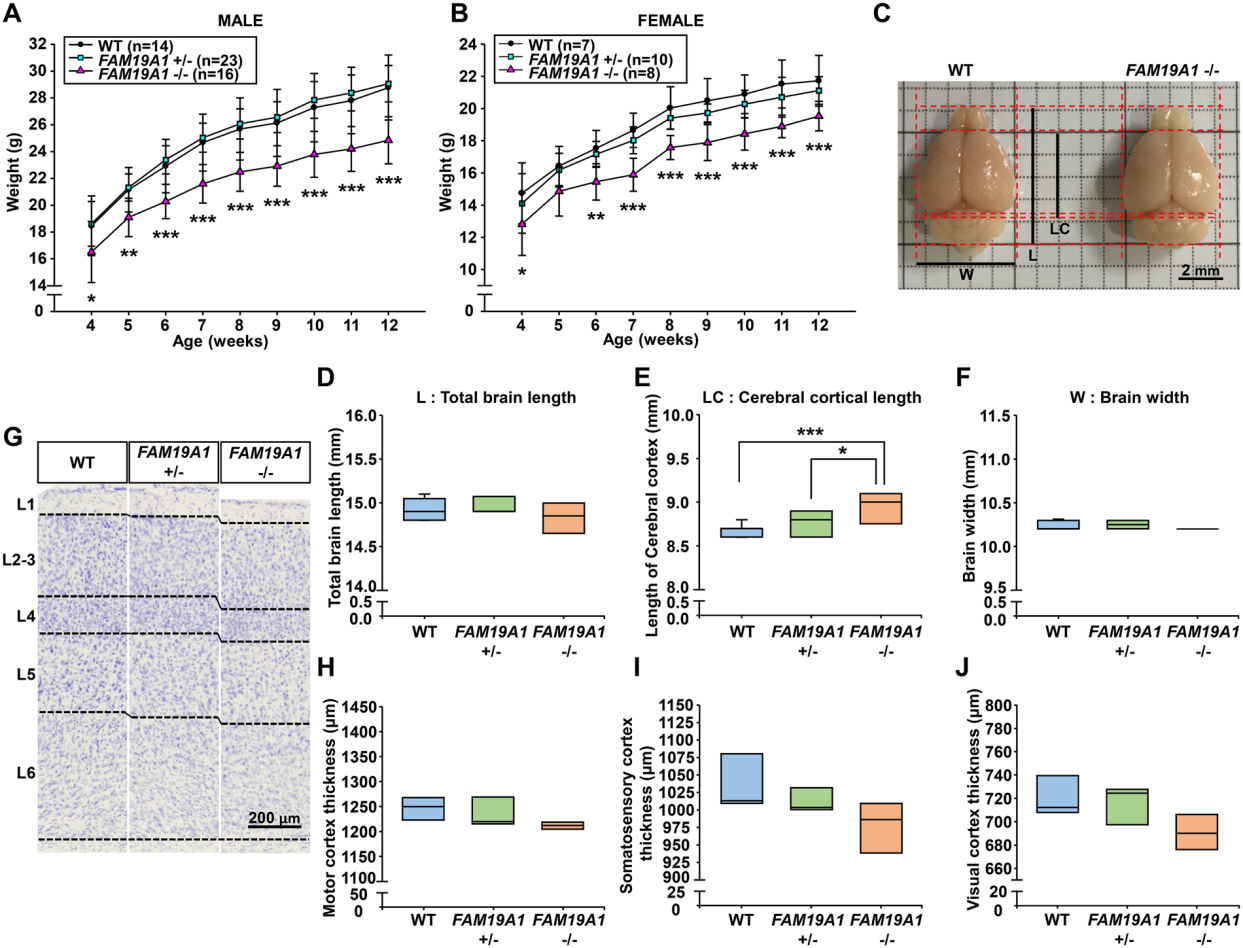
Differences in body weight and gross brain size between adult WT and *FAM19A1 LacZ* knock-in (KI) mice. (**A**) Changes in body weight with age in male *FAM19A1 LacZ* KI mice; F(2, 450) = 189.5, *p* < 0.0001 across genotypes. (**B**) Changes in body weight with age in female *FAM19A1 LacZ* KI mice; F(2, 198) = 71.07, *p* < 0.0001 across genotypes. (**C**) Whole-mount view of the WT and *FAM19A1* (−/−) adult mouse brains. (**D**–**F**) Total brain length, cerebral cortical length and brain width, respectively, of WT (n = 9), *FAM19A1* +/− (n = 8) and *FAM19A1* −/− (n = 8) adult mouse brains. For panel E, F(2, 22) = 10.04, *p* = 0.0008. (**G**) Motor cortical layers in the Nissl-stained brain tissues of WT and *FAM19A1 LacZ* KI mice. (**H**–**J**) Cortical thickness of the motor (F(2, 11)= 2.363, *p* = 0.1401), somatosensory (F(2, 11) = 3.652, *p* = 0.0608) and visual cortex (F(2, 11) = 3.911, *p* = 0.0521), respectively, of WT (n = 5), *FAM19A1* +/− (n = 5) and *FAM19A1* −/− (n = 4) adult mice. Scale bars represent 2 mm in panel C and 200 μm in panel G. Data are presented as means ± standard errors of the means (SEM). *p < 0.05, **p < 0.01, ***p < 0.001 versus WT or *FAM19A1* +/− by one-way or two-way analysis of variance (ANOVA) with Bonferroni *post hoc* tests.

For general features, *FAM19A1* −/− mice were born with approximately 24–25% of Mendelian frequency and similar sex-ratio from the heterozygous parents (Supplementary Fig. S6B). There were no notable differences in the gross appearance between newborn genotypes immediately after birth; however, it was observed that *FAM19A1* −/− mice were weighed significantly less than WT mice (Fig. 4A,B).

The total length and width of adult brains were similar between WT and *FAM19A1* −/− mice (Fig. 4C,D,F), whereas the cerebral cortical length was longer in *FAM19A1* −/− mice compared to WT mice (Fig. 4E). Ablation of genes specifically expressed in the cortical layer often leads to improper cortical layer assembly^32^. However, cerebral cortical volume remained unaffected (Supplementary Fig. S7). In addition, there were no notable structural abnormalities in brain architecture detected by gross observations on the X-gal stained brain sections of *FAM19A1* −/− mice (data not shown). The thickness of all neocortical areas was not significantly reduced in *FAM19A1* −/− mice (Fig. 4H,I,J). In terms of cortical layer proportion, however, L4 in visual cortex and L6 in motor cortex were decreased in *FAM19A1* −/− compared to WT mice (Supplementary Fig. S8).

While these changes in cortical layer thickness could be the result of abnormal cytoarchitecture, no significant differences in the neuronal and glial cell populations of the cortical layers were observed between the *FAM19A1* −/− and WT mice (Supplementary Figs. S9 and S10). In addition, there were no notable abnormalities in morphology of neuronal and glial cells (Data not shown). Taken together, these findings indicate that global *FAM19A1* ablation reduced body weight gain and mildly altered the neocortical architecture but did not significantly impact cell type composition of the cortex.

### FAM19A1-deficient mice displayed hyperactive behavior

**Figure 5:**
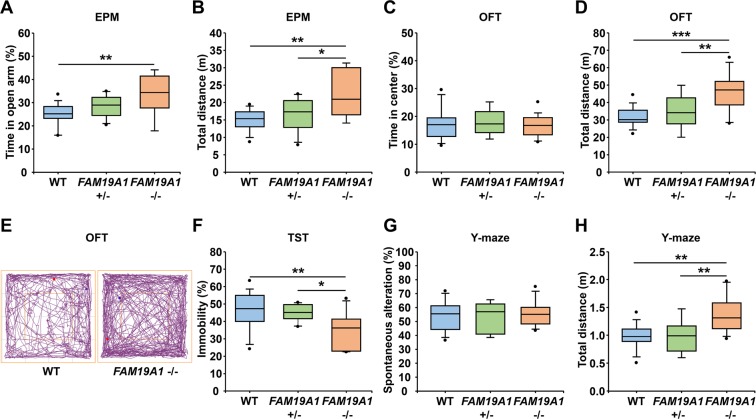
Hyperactive behavior in *FAM19A1* −/− mice. (**A**) Time in the open arm (F(2, 32) = 6.469, *p* = 0.0044) and (**B**) total distance (F(2, 32) = 7.803, *p* = 0.0017) were measured in the elevated plus maze (EPM) test in WT (n = 15), *FAM19A1* +/− (n = 11) and *FAM19A1* −/− (n = 9) mice. (**C**) Time in the center and (**D**) total distance (F(2, 43) = 14.50, *p* < 0.0001) were measured in the open field test (OFT) in WT (n = 19), *FAM19A1* +/− (n = 9) and *FAM19A1* −/− (n = 18) mice. (**E**) Simple line tracings of movements in the OFT arena. (**F**) Percentage of immobile time (F(2, 36) = 5.478, *p* = 0.0084) on the tail suspension test (TST) in WT (n = 17), *FAM19A1* +/− (n = 11) and *FAM19A1* −/− (n = 11) mice. (**G**) Spontaneous alteration and (H) total distance (F(2, 34) = 8.257, *p* = 0.0012) on the Y-maze in WT (n = 16), *FAM19A1* +/− (n = 9) and *FAM19A1* −/− (n = 12) mice. Data are presented as means ± standard errors of the means (SEM). *p < 0.05, **p < 0.01, ***p < 0.001 vs WT or *FAM19A1* +/− by one-way analysis of variance (ANOVA) with Bonferroni *post hoc* tests.

Several behavior tests were performed in *FAM19A1 LacZ* KI mice to investigate the effect of FAM19A1 depletion on behavior. *FAM19A1* is expressed in several areas of the limbic system including the prelimbic cortex and the amygdala (Fig. 3C,G), which are known to be involved in emotional processing^33^. To evaluate the effects of FAM19A1 depletion on anxiety and depression, the elevated plus maze (EPM) test, the open field test (OFT), and the tail suspension test (TST) were performed. On the EPM test, the amount of time spent in the open arms (Fig. 5A) and the total distance travelled increased (Fig. 5B) in *FAM19A1* −/− mice compared to WT mice. On the OFT, the amount of time spent in the center of the OFT arena was similar between *FAM19A1* −/− and WT mice, but the total distance travelled was higher in *FAM19A1* −/− mice (Fig. 5C,D,E). On the TST, *FAM19A1* −/− mice displayed lower immobility than WT mice (Fig. 5F). It should be noted that *FAM19A1* −/− mice consistently exhibited a significant increase in basal locomotor activity than WT mice. Thus, anxiety and depression were unable to be determined in these behavior tests. These findings indicate that FAM19A1 ablation is associated with hyperactive behavior.

### Long-term memory deficits in *FAM19A1* −/− mice

Short-term memory (STM), and in particular spatial working memory, is known to involve an interaction between CA1 and CA3 of the hippocampus and the CEn^34^, where FAM19A1 is highly expressed (Fig. 3F,J). On the Y-maze, a spatial working memory test, no significant differences in spontaneous alteration were observed between *FAM19A1* −/− and WT mice (Fig. 5G). However, the total distance travelled was significantly increased in *FAM19A1* −/− mice, which is consistent with findings on the EPM and OFT, confirming the presence of hyperactive behavior in *FAM19A1* −/− mice (Fig. 5H).

**Figure 6:**
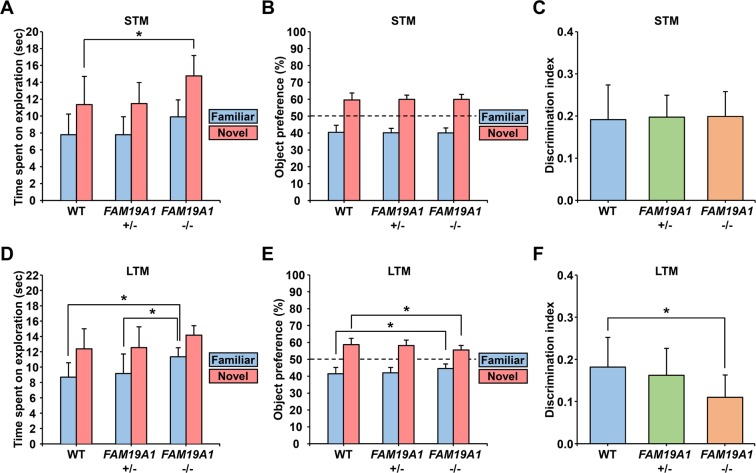
Short-term and long-term memory in *FAM19A1* −/− mice. (**A**) Time spent on exploration (F(2, 29) = 5.088, *p* = 0.0128), (**B**) object preference and (**C**) the discrimination index on the short-term memory novel object recognition (NOR) test in WT (n = 13), *FAM19A1* +/− (n = 8) and *FAM19A1* −/− (n = 11) mice. (**D**) Time spent on exploration (F(2, 37) = 5.159, *p* = 0.0106), (**E**) object preference (F(2, 38) = 5.531, *p* = 0.0078) and (**F**) the discrimination index (F(2, 37) = 3.870, *p* = 0.0298) on the long-term memory NOR test for WT (n = 11), *FAM19A1* +/− (n = 18) and *FAM19A1* −/− (n = 11) mice. Data are presented as means ± standard errors of the means (SEM). *p < 0.05, **p < 0.01, ***p < 0.001 versus WT or *FAM19A1* +/− by one-way analysis of variance (ANOVA) with Bonferroni *post hoc* tests.

Novel object recognition (NOR) tests were performed to examine possible deficits in object recognition memory. On the short-term memory version of the NOR test, there was no significant difference in preference towards novel objects between *FAM19A1* −/− and WT mice (Fig. 6B,C). However, on the long-term memory (LTM) version of the NOR test, *FAM19A1* −/− mice exhibited lower preference towards novel objects and higher preference for familiar objects than WT mice (Fig. 6E,F). These findings indicate that *FAM19A1* −/− mice had lower ability to discriminate between familiar and novel objects 24 hours after acquisition, indicating possible deficits in LTM in *FAM19A1* −/− mice. In addition, *FAM19A1* −/− mice tended to spend more time exploring the objects, regardless of whether the object was novel or familiar, than WT mice (Fig. 6A,D). This finding may be related to the hyperactive behavior of *FAM19A1* −/−mice, as they may encounter the objects more frequently.

### Impairment of conditioned fear acquisition in *FAM19A1* −/− mice

**Figure 7:**
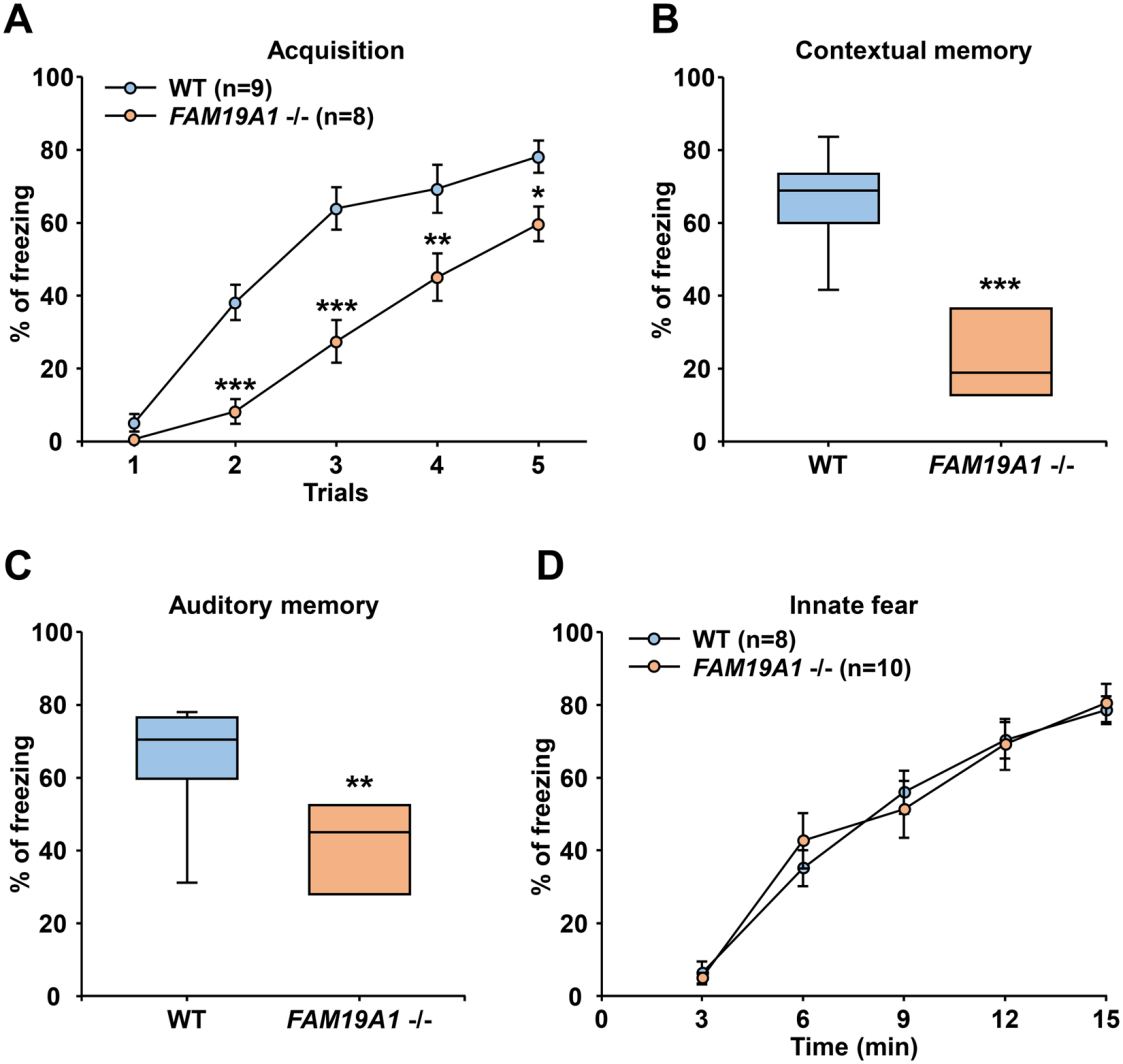
Fear responses in *FAM19A1* −/− mice. (**A**–**C**) Pavlovian fear conditioning test. (**A**) Fear conditioning during the acquisition phase; F(1, 85) = 53.68, *p* < 0.0001 across genotypes. (**B**) Contextual and (**C**) auditory memory tests were performed 24 h after the acquisition phase in WT (n = 9), and *FAM19A1* −/− (n = 8) mice. (**D**) The innate fear test was performed using synthetic fox feces odor, 2,5-dihydro-2,4,5-trimethylthiazoline (TMT) in WT (n = 8) and *FAM19A1* −/− (n = 10) mice. Data are presented as means ± standard errors of the means (SEM). *p < 0.05, **p < 0.01, ***p < 0.001 versus WT by two-way analysis of variance (ANOVA) with Bonferroni *post hoc* test or Student’s *t* test.

A Pavlovian fear conditioning test was employed to investigate the fear response in *FAM19A1* −/− mice. *FAM19A1* is expressed in fear processing-related areas such as the amygdala and the hippocampus (Fig. 3F,G)^35^, which suggests that FAM19A1 may be involved in fear processing. During the fear acquisition phase, *FAM19A1* −/− mice exhibited less freezing behavior than WT mice (Fig. 7A) such that fear conditioning was not properly induced; *FAM19A1* −/− mice therefore also exhibited less freezing behavior during the subsequent contextual and auditory memory tests (Fig. 7B,C).

It was hypothesized that this lack of fear acquisition in *FAM19A1* −/− mice might be related to the innate fear response. In general, mice innately experience fear when they encounter the smell of their predator’s odor. To test this innate fear response, *FAM19A1* −/− and WT mice were exposed to 2,5-dihydro-2,4,5-trimethylthiazoline (TMT), a component of fox feces. *FAM19A1* −/− mice exhibited the same magnitude of fear response as WT mice over the entire TMT exposure (Fig. 7D), indicating that the innate fear response remained intact in *FAM19A1* −/− mice. This finding suggests that the inability of *FAM19A1* −/− mice to acquire a conditioned fear response is not related to the innate fear response, but instead may involve other mechanisms such as sensory dysfunctions that prevent *FAM19A1* −/− mice from forming an association between conditioned and unconditioned stimuli.

## Discussion

FAM19A1 is an evolutionarily conserved secretory polypeptide, which implies that FAM19A1 plays a critical role in the CNS, where it is primarily expressed^17^. In this study, a knock-in mouse expressing β-galactosidase via the *FAM19A1* promotor was utilized to map the expression pattern of *FAM19A1* to understand the putative functions of FAM19A1 in both developing and mature brains. *FAM19A1* was expressed in the brain starting during the embryonic stage and continued to be expressed in postnatal and adult brains. In the adult brain, *FAM19A1* was expressed in the pyramidal neurons across the entire neocortex and in various limbic regions. In addition, mice deficient in FAM19A1 exhibited hyperactive behavior and long-term memory deficits and were unable to acquire a conditioned fear response. These findings indicate that FAM19A1 may play important roles in both brain development and function.

During the CNS developmental stages, *FAM19A1* expression was observed in the CPf as early as E14.5. As the brain matured, *FAM19A1* expression gradually expanded to the entire neocortex and at the late development, it was also observed in the cingulate cortex (Cg) and the retrosplenial cortex (RSC). The CPf is known to mature faster than other cortical areas whereas the Cg and the RSC are known to mature at relatively later stages of CNS development^36,37^, suggesting that FAM19A1 may be related to the structural maturation of the developing brain. The postnatal period is when neurogenesis and neuronal migration are almost completed and cortical layer development and synaptogenesis are actively in progress^38^. Cortical layers are constructed in the inside-out manner^39^ and *FAM19A1* expression was observed earlier in neurons of L5 compared to neurons of L2-3. Thus, it is assumed that FAM19A1 is expressed in the neurons that are undergoing the maturation process.

In the anxiety-related amygdala circuitry, the basolateral nuclei receive incoming stimuli from other limbic areas, including the prefrontal cortex and the thalamus. This information is then sent to the central nucleus of the amygdala (CeA) via glutamatergic connections for further processing^40^. *FAM19A1* was expressed exclusively in the basolateral nuclei, and the majority of neurons in these nuclei are pyramidal efferent neurons^41^; thus, FAM19A1 may be mainly secreted by excitatory pyramidal neurons. Moreover, the cortical layer-specific gene expressions are mostly found among the neurons and these molecular identities are often related to the neuronal types^39^. In this study, *FAM19A1* expressing cortical cell types were identified to be the pyramidal neurons and this finding is consistent with open-source data from single cell-based RNA-sequencing of WT mouse brain, which reports that the majority of *FAM19A1*-expressing cell types are excitatory neurons^28^.

The brain receives sensory inputs and processes them into a construct that can be utilized as information via neuronal circuits. Based on the expression pattern of FAM19A1 in the adult mouse brain, FAM19A1 may participate in several sensory information transducing processes. In the olfaction circuit, *FAM19A1* was expressed in most of the olfactory-related brain regions, including the AO, the CPf, the cortical amygdala and the lateral CEn, but was not expressed in the olfactory bulb, where sensory information is initially received^42^. For the visual circuit, *FAM19A1* was expressed in the Op, an intermediate zone where axons from the optic tract meet the visual cortical connection, but was not expressed in the optic tract itself^43^. Moreover, in the auditory circuit, *FAM19A1* was identified in the DC, where all auditory nerve fibers from the cochlea form their first synapses^44^. These findings imply that FAM19A1 may not be involved in processes related to receiving sensory stimuli, but instead may be related to intermediate information transduction or information processing.

One prominent feature of *FAM19A1* −/− mice was low body weight compared to WT mice. There was no notable difference in the gross appearance between newborn *FAM19A1* −/− and WT mice. Thus, *FAM19A1* −/− mice were able to develop normally in the uterus of *FAM19A1* +/− mothers with WT siblings. One possible explanation for low body weight in *FAM19A1* −/− mice may be abnormal dietary and energy balance. Recently, alteration of food intake behavior was reported in male *FAM19A1* ablated mice, suggesting a potential role of FAM19A1 as a metabolic neurokine in a sex-dependent manner^45^. *FAM19A1* was expressed in the VMH which is known to be involved in food intake behaviors and energy homeostasis^46^. The neurons in the VMH are predominantly glutamatergic^47^, and it has been reported that defects in the glutamatergic leptin receptor-expressing neurons of the VMH are associated with weight change due to thermogenic-related altered energy expenditure^47^. Thus, low body weight in *FAM19A1* −/− mice may be related to the abnormal activity of these glutamatergic neurons in the VMH. Moreover, the hyperactive behavior observed in *FAM19A1* −/− mice, which requires more energy, may contribute to low body weight.

The confinement of *FAM19A1* expression to the limbic system suggests that FAM19A1 may be important to limbic system-related functions, such as emotional and memory processing^33^. According to previous studies, the long-term potentiation of synaptic plasticity in the basolateral amygdala is important for stabilization of fear memory consolidation^48^. The ablation of FAM19A1, which is expressed in the basolateral amygdala, resulted in an inability to acquire a conditioned fear response. One explanation is that FAM19A1 may play a role in maintaining synaptic plasticity in this region. Another explanation is that FAM19A1-deficient mice could not acquire the fear response due to impairments in the auditory or other sensory systems, as *FAM19A1* was also expressed in the DC and dorsal root ganglia (Supplementary Fig. S5F,N). Additional phenotypic analysis of *FAM19A1* −/− mice is needed, including hearing and other sensory tests. In addition to that, synaptic plasticity in *FAM19A1* −/− mice needs to be investigated.

Memory is a fundamental brain function and is controlled mainly by the limbic system. Once external information is received, it is initially encoded as STM. This STM is then converted into LTM via memory consolidation^49^. In *FAM19A1* −/− mice, STM formation seems to be remained intact, but LTM appeared to be affected, as *FAM19A1* −/− mice were unable to distinguish novel objects from familiar objects. This finding implies that FAM19A1 may not be involved in the memory encoding process, but instead may participate in the memory consolidation process to generate LTM. Memory consolidation requires changes in synaptic remodeling, which leads to the long-term potentiation that maintains synaptic plasticity for memory formation^50^. The absence of FAM19A1 may contribute to abnormalities in synaptic function, resulting in a failure to form LTM. This hypothetical explanation suggests that FAM19A1 may be involved in the long-term potentiation process.

Another behavioral phenotype displayed by *FAM19A1* −/− mice was hyperactivity. Hyperactivity is closely linked to the human neurodevelopmental disorder called, ADHD. Although the pathophysiological and molecular mechanisms of ADHD remain poorly understood, there have been several studies suggesting that patients with ADHD have compromised brain connectivity, particularly in the corpus callosum and the corticospinal tract, which contributes to dysfunctional communication in the superior longitudinal fasciculus and the cortico-limbic areas^51,52^. *FAM19A1* was expressed in the cortical L5 pyramidal neurons as well as the cortico-spinal tract across the entire neocortex, which project their axons to other brain areas to form several crucial brain connections. This expression pattern implies that the absence of FAM19A1 may alter the functional plasticity of the neurons, which may lead to dysfunctional brain connectivity resulting in hyperactive behavior.

In the open-source collective RNA-sequencing database, ARCHS4 (All RNA-seq and ChIP-seq sample and signature search), the highest predicted biological function of FAM19A1 is presynaptic and/or postsynaptic membrane assembly, and the highest predicted mouse phenotypes associated with FAM19A1 were abnormal central patterns, synaptic plasticity and abnormal learning and memory^53^. Some of these predicted biological functions and phenotypes are consistent with the phenotypes observed in *FAM19A1* −/− mice in this study at limited extend. In particular, abnormal synaptic plasticity may be associated with the hyperactive behavior and long-term memory deficits we observed in *FAM19A1* −/− mice^50,54^. In the genome-wide association for human genetic variants database, GWASdb, FAM19A1 was identified to be associated with ADHD^55^. In addition, there have been human case reports that patients with 3p14.1 interstitial deletion, where the *FAM19A1* gene is located, exhibited intellectual disability, and in some cases, autistic features^56,57^. Although this chromosomal position also contains other neurodevelopment-related genes, such as FOXP1, it is still rationally deduced that the ablation of FAM19A1, starting from the developmental stages may contribute to hyperactive symptom generation.

In summary, as a CNS-specific secretory polypeptide, the *FAM19A1* gene was expressed starting in the early embryonic period within restricted brain areas and gradually expanded to all neocortical areas as well as areas of the limbic system during the postnatal period. Based on this expression pattern, the major *FAM19A1*-expressing cell type was suspected to be excitatory pyramidal neurons. In addition, FAM19A1-deficient mice exhibited hyperactive behavior, long-term memory deficits, and an inability to acquire a conditioned fear response, suggesting that FAM19A1 may be involved in synaptic plasticity and long-term potentiation. Given the involvement of FAM19A1 in human case reports of neurodevelopmental disorders, we conclude that FAM19A1 may play important roles in both neurodevelopment and brain function.

## Materials and Methods

### Animals and handling

All mice were housed in a temperature-controlled (22~23 °C) facility under a 12:12 light:dark photoperiod (lights on at 8:00am), with ad lib access to standard mouse chow and water. All animal procedures were approved by the Institutional Animal Care and Use Committee (IACUC) of Korea University (KOREA-2016-0091) and performed in accordance with guidelines and regulations of IACUC of Korea University.

The *FAM19A1 LacZ* knock-in (KI) mouse was generated at the University of California, Davis, Mouse Biology Program (MBP)^58^. In brief, the *LacZ* sequence containing the target vector (Fig. 1A) was constructed and inserted into embryonic stem (ES) cells via electroporation. Target vector incorporation was validated by genotyping and chromosome counting of the transgenic ES cells. The confirmed transgenic ES cells were injected into blastocysts and transferred to the uterus of a female recipient mouse. A germline transmission test was performed to confirm chimeric generation. The generated *FAM19A1 LacZ* KI chimeric mice were backcrossed onto C57BL/6J genetic background. The *FAM19A1 LacZ* KI strain was maintained by mating heterozygous *FAM19A1 LacZ* KI male mice with WT C57BL/6J female mice (Maine, United States). To obtain homozygous *FAM19A1 LacZ* KI mice, heterozygous *FAM19A1 LacZ* KI male mice were mated with heterozygous *FAM19A1 LacZ* KI female mice. Genotyping was performed using the following primers: FWT dn1: 5′ TCG CAC AAG CAC TTA TCC AC 3′, FKI dn2: 5′ ATC TGA GTT GCT GGC TTG GT 3′ and F UP1: 5′ AGC TTC TGG GAA AGG TCT TCA 3′.

### X-gal staining for embryonic and postnatal brains

X-gal staining was conducted as previously described^59^. For embryonic X-gal staining, pregnant mice were sacrificed by cervical dislocation and the embryos were isolated. Whole embryos at E12.5 were fixed in 4% PFA and 0.2% glutaraldehyde (GTA) in PBS for 15 min at 4 °C. For embryos and postnatal mice older than E14.5, the heads were cut and the skins were removed. The heads of the embryos were fixed for 1~2 h at 4 °C. The postnatal brains were isolated from the skulls and fixed for 1~2 h at 4 °C. The fixed tissues were then washed with PBS twice for 5 min and incubated in X-gal staining solution (1 mg/ml of X-gal, 2 mM MgCl_2_, 5 mM EGTA, 5 mM potassium ferrocyanide, 5 mM potassium ferricyanide, 0.01% sodium deoxycholate, and 0.02% Nonidet-P40 in 0.1 M phosphate buffer (PB) at pH 7.3) for 24–48 h in darkness at 37 °C. The stained tissues were post-fixed with 4% PFA in PBS overnight at 4 °C, and washed with PBS, then whole brain images were obtained. The stained whole brains were cryo-protected with 30% sucrose in phosphate-buffered saline (PBS). and cut into 40-μm sections using Cryostat (Leica, Wetzlar, Germany). Nuclear Fast Red (H-3403, VECTOR, California, United States) was used as a counter-stain where appropriate. Images of the sections were taken using a slide-scanner (Axio scan Z1, Zeiss, Oberkochen, Germany).

### X-gal staining for adult brains, spinal cords and dorsal root ganglia

X-gal staining was performed as previously described^59^. Adult mice (8–12 weeks old) were perfused with 4% PFA and 0.2% GTA in PB. The brains, spinal cords and dorsal root ganglia were isolated and post-fixed in 0.2% GTA in PB for 12 h at 4 °C.The tissues were then cryo-protected in 30% sucrose in PBS and serially cross-sectioned into 40-μm sections using Cryostat (Leica). The sectioned tissues then were incubated in the X-gal staining solution for 24–48 h at 37 °C in darkness. Images of the sections were taken using a slide-scanner (Axio scan Z1, Zeiss).

### Immunohistochemical analysis

Animals were perfused with 4% paraformaldehyde (PFA) in (PBS). The isolated brains were post-fixed overnight. The brains were then cryo-protected in 30% sucrose in PBS and serially cross-sectioned into 40-μm sections using Cryostat (Leica) The sections were blocked with 3% bovine serum albumin (BSA) and 0.1% Triton X-100 in PBS for 30 min at room temperature. Primary antibodies were applied overnight at 4 °C and then the appropriate fluorescent conjugated secondary antibodies were applied with Hoechst 33342 (Invitrogen, California, United States) for 30 min at room temperature. For immunohistochemistry on X-gal stained tissues^59,60^, the brain tissues were initially stained in X-gal staining solution and then washed in PBS. Normal immunohistochemistry procedures were carried out with X-gal stained tissues. For image acquisition, X-gal precipitates were excited at 633 nm and the fluorescence signals were detected at 650–770 nm. The following antibodies were used: β-galactosidase (1:500; ab9391, abcam, Cambridge, United Kingdom); anti-CTIP2 (1:500; ab18465, abcam); anti-CUX1 (1:500; SC-13024, Santa Cruz, Texas, United States); anti-GFAP (1:500; Z0334, DAKO, California, United States); anti-Iba1 (1:500; 019-19741, Wako, Osaka, Japan); anti-Olig2 (1:500; AB9610, Millipore, Massachusetts, United States); and fluorescent conjugated anti-rabbit, anti-rat and anti-chick (1:500; Life technologies, California, United States). Images were obtained using a confocal microscope (TCS SP8, Leica).

### Nissl staining

Animals were perfused with 4% PFA in PBS. The brains were isolated and post-fixed overnight. The brains were then cryo-protected in 30% sucrose in PBS and serially cross-sectioned into 40-μm sections using Cryostat (Leica). The brain sections were dipped in a Nissl stain solution, 1% w/v Cresyl Violet (C5042, Sigma-Aldrich, Missouri, United states) and 1% v/v glacial acetic acid at 50 °C for 5 to 10 min. After a wash with distilled water, the tissues were differentiated in ethyl alcohol for 10 to 20 min. The dehydrated tissues were cleared in xylene and mounted with Permount (SP15-500, Thermo Fisher Scientific, Massachusetts, United States). The images of the sections were taken using a slide-scanner (Axio scan Z1, Zeiss).

See the Supplementary Information for details on the generation of the polyclonal anti-FAM19A1 antibody, western blot analysis, reverse transcription PCR^61^, *in situ* hybridization, body weight measurement, whole-brain size measurement, cerebral cortex analysis with unbiased stereology^62^, cortical thickness measurement^62,63^ and neuronal and glial cell density analysis^62,63^.

### Behavior analysis

Before commencing the behavioral tests, all male mice (8–12 weeks old) were handled for 5 min daily for 3 days to habituate them to the hands of the test instructor. See the Supplementary Information for details on the elevated plus maze (EPM) test, open field test (OFT), tail suspension test (TST), Y-maze, novel object recognition (NOR) test, Pavlovian fear conditioning and unconditioned innate fear response, which were performed as previously described^64^.

### Statistical analysis

All statistical analysis was performed using GraphPad Prism 5 (GraphPad Software Inc., California, United States) and the data are shown as means ± standard errors of the means (SEM). Statistical significance was evaluated using Student’s *t* tests and/or one-way or two-way analysis of variance (ANOVA) with Bonferroni *post hoc* tests. A *p-*value less than 0.05 was considered statistically significant.

## Supplementary information

Supplementary Information.

## Data Availability

All data are available from the GPCR laboratory of Korea University, and there are no restrictions on data availability.
