# Supplementary material for: The unique expression profile of FAM19A1 in the mouse brain and its association with hyperactivity, long-term memory and fear acquisition
Source: Sci Rep. 2020 Mar 2;10:3969. doi: 10.1038/s41598-020-60266-1 (PMC7052240; doi:10.1038/s41598-020-60266-1)
Supplement: Supplementary file 1 — Supplementary Information. [file 41598_2020_60266_MOESM1_ESM.pdf]

The unique expression profile of *FAM19A1* in the mouse brain and its association with hyperactivity, long-term memory and fear acquisition

Hyo Jeong Yong[1], Nui Ha[2], Eun Bee Cho[2], Seongsik Yun[1], Hyun Kim[3], Jong-Ik Hwang[1,*], Jae Young Seong[1,*]

[1]The GPCR laboratory, Graduate School of Biomedical Science, Korea University College of Medicine, Seoul 02841, Republic of Korea
[2]Neuracle Science Co. Ltd., Seoul 02841, Republic of Korea
[3] Department of Anatomy, Korea University College of Medicine, Seoul 02841, Republic of Korea

*Corresponding authors: Jae Young Seong (Email: jyseong@korea.ac.kr) and Jong-Ik Hwang (Email: hjibio@korea.ac.kr)
Graduate School of Biomedical Science, Korea University College of Medicine, Seoul 02841, Republic of Korea.
Tel: 82-2-2286-1090, 1093; Fax: 82-2-921-4355

Supplementary Information

Materials and methods

Generation of polyclonal anti-FAM19A1 antibody

Polyclonal anti-FAM19A1 antibody was obtained from the antisera of rabbits immunized with emulsions containing keyhole limpet hemocyanin (KLH) or ovalbumin (OVA) conjugated synthetic FAM19A1 peptides, CHGSLQHTFQQHHLHRPEGG (Anygen, Gwangju, South Korea) and complete or incomplete Freund's adjuvant (F5881 and F5506 respectively, Sigma-Aldrich, Missouri, United states). An immunoglobulin G (IgG) fraction was purified from immunized rabbit sera using Protein A Sepharose (IPA-300, Repligen, Massachusetts, United States). For validation of in-house generated FAM19A1 antibody, Flag tagged FAM19A family proteins were overexpressed in HEK 293 cells using Lipofectamine® 2000 Transfection Reagent (Invitrogen, California, United States). Cell lysates were collected and applied for western blot analysis.

Western blot analysis

Brain regions of adult mice were isolated and lysed with a buffer containing 50 mM Tris-HCL (pH7.5), 0.2% sodium dodecyl sulfate, 1% Triton X-100, 0.25% sodium deoxycholate, 10 mM sodium fluoride, 1 mM ethylenediaminetetraacetic acid, 150 mM sodium chloride and a protein inhibitor cocktail tablet (Roche Applied Science, Penzberg, Germany). Protein contents in the lysates were quantified using Bio-Rad Bradford protein assay reagent (Bio-Rad, California, United States) and resolved on a NuPAGE™ 4-12% Bis-Tris gel (Invitrogen). The resolved proteins were transferred to Trans-Blot Turbo Mini PVDF (Bio-Rad) using Trans-Blot Turbo Transfer System (Bio-Rad), and the blots were blocked in Tris-buffered saline containing 0.3% Tween 20 and 5% skim milk for 30 min at room temperature. The blots were incubated with primary antibodies for 3 h and then incubated with horseradish peroxidase-conjugated secondary antibodies for 1 h at room temperature. After application of enhanced chemiluminescence (ECL) reagents (RPN2232, GE Healthcare, Illinois, United States), immunoreactive bands were visualized by exposing the blots to X-ray film. The following antibodies were used: rabbit polyclonal anti-FAM19A1 (1:1000; generated in-lab), anti-Flag (1:500; F7425, Sigma-Aldrich), anti-β-actin (1:2000; ab8227, abcam, Cambridge, United Kingdom), and HRP conjugated anti-rabbit (1:5000; Jackson ImmunoResearch Laboratories, Pennsylvania, United States).

RNA isolation and Reverse transcription PCR (RT-PCR)

Total RNAs were isolated from brain regions of adult mouse by single-step acid guanidinium thiocyanate-phenol-chloroform method[61]. For 1 µg of each RNA sample was reverse-transcribed with M-MLV reverse transcriptase (M1707, Promega, Wisconsin, United States). Then, aliquots of the cDNAs were subjected to conventional PCR with following primers, mFAM19A1_F, 5'-ATG GCA ATG GTC TCT GCA-3', mFAM19A1_R, 5'-TTA GGT TCT TGG GTG AAT-3', mGAPDH_F, 5'-CAT CCA

CTG GTG CTG CCA AGG CTG T-3’ and mGAPDH_R, 5’-ACA ACC TGG TCC TCA GTG TAG CCC A-3’. Annealing temperature was 57 °C and 25 reaction cycles were employed.

In situ hybridization

Frozen rat brains were sliced into 20-mm sections on the coronal plane using a Cryostat (Leica, Wetzlar, Germany) and directly thaw-mounted onto TESFA-coated glass slides. For fixation, 4% paraformaldehyde (PFA) in phosphate-buffered saline (PBS) was applied to the slices and acetylated with 0.25% acetic oxides dissolved in a mixture of 1 M triethanolamine (TEA) and normal saline (pH 8.0). Subsequently, the sections were hybridized overnight with radio-labeled rat FAM19A1 cRNA probes, FAM19A1-F, 5'-ATG ACA ATG GTC TCT GCA-3' and FAM19A1-R, 5'-TTA GGT TCT TGG GTG AAT-3' (1.2x106 cpm). After washing with 2× saline-sodium citrate (SSC) buffer, the sections were treated with RNase A (20 mg/ml) for 30 min at 37°C. The sections were rinsed with SSC buffer and again dipped into the 0.1× SSC buffer at 60°C. The samples were dehydrated and exposed to the X-ray film (Biomax MR, Kodak, New York, United States).

Body weight measurement

Body weights of male and female wild type (WT), heterozygous *FAM19A1 LacZ* knock-in (KI) and homozygous *FAM19A1 LacZ* KI mice were measured for 8 weeks starting from postnatal week 4.

Whole-brain size measurement

Animals were perfused with PBS and then the brains were isolated. The total length, cerebral cortical length and width of the brains were measured with a digital caliper.

Cerebral cortex analysis with unbiased stereology

For neuronal cell detection on brain tissues, immunoenzyme staining was used. Anti-NeuN (1:500; ab128886, abcam) was used as primary antibody and biotinylated anti-rabbit (1:500, BA-1000, VECTOR, California, United States) and DAB Peroxidase (HRP) Substrate Kit (SK-4100, VECTOR) were employed. The immunostained brain tissues were visualized with motorized stage equipped light microscope (Zeiss, Oberkochen, Germany) and total neuronal cell and the volume of cerebral cortex were determined using Stereo Investigator 7 (MBF Bioscience, Vermont, United States). Cerebral cortical areas were defined using the mouse brain atlas[62]. Total of 13 brain sections in thickness of 40 μm were examined with section periodicity of 12 for each genotype (n = 5). For stereological probe, Optical Disector was used. Counting frames (50 x 50 x 32 μm) were placed on sampling grid matrix (500 x 500 μm) by the program. All obtained measurements had Gundersen coefficients of error for m = 1 less than 0.10.

Cortical thickness measurement

For the cortical thickness measurement, cortical areas were defined using the mouse brain atlas (motor cortex, interaural 5.14~4.78 mm, Bregma 1.34~0.98 mm; somatosensoty cortex, interaural 2.58~2.22 mm, Bregma -1.22~-1.58 mm; visual cortex, interaural 1.26-0.88 mm, Bregma -2.54~2.92 mm)[59]. For each genotype (n = 4-5), three non-adjacent brain sections (interval of 120 μm) were Nissl-stained and the images were taken using a slide-scanner (Axio scan Z1, Zeiss). Cortical layers were distinguished according to the cellular morphology and organization as illustrated previously[62,63]. Average cortical thickness of each section was obtained from at least three measurements using the ZEN program (Zeiss).

Neuronal cell density analysis

Unbiased stereological neuronal cell density measurement was performed on each motor cortical layer. For neuronal cell detection on brain tissues, immunoenzyme staining with anti-NeuN was employed as described in cerebral cortex analysis method. The neuronal cell density was determined using Stereo Investigator 7 (MBF Bioscience). Each cortical layer in the motor cortex was determined based on the neuronal morphology[62, 63]. The brain section thickness was 40 μm and total of three sections were examined with section periodicity of three for each genotype (n = 4). For stereological probe, Optical Disector was used. Counting frames (25 x 25 x 32 μm) were placed on sampling grid matrix (for cortical layer 1 and 4; 100 x 100 μm and for cortical layer 2-3, 5 and 6; 200 x 200 μm) by the program. All obtained measurements had Gundersen coefficients of error for m = 1 less than 0.10.

Glial cell density analysis

For the glial cell density in the cortical layer, three non-adjacent brain sections (interval of 120 μm) were immunostained with appropriate markers (GFAP for astrocytes, Iba1 for microglia and Olig2 for oligodendrocytes), and those cells that were co-localized with Hoechst signal were counted using LAS X (Leica). Three measurement were made on each section and the obtained values were averaged. At least four mice from each experimental group were used for these quantification analyses.

Elevated plus maze (EPM) test

The elevated plus maze (EPM) had four perpendicular arms, two open (5 x 30 cm) and two closed (5 x 30 cm), with 20-cm high walls. The maze was elevated 50 cm above the ground. The test animals were individually placed in the center of the maze facing one of the open arms and allowed to freely explore for 15 min. Recorded videos were analyzed by the ANY-maze video tracking program (Stoelting, Illinois, United States). The number of open arm entries, time spent in the open arms, number of center crossings and the total distance travelled were recorded. An entry was defined as the placing of all four paws within the arm.

Open field test (OFT)

The w40 x h40 x d40 cm OFT apparatus was made of opaque plastic. The test arena was defined as 30% of the central zone and the surrounding border zone. The test animals were individually placed in the center of the arena and their behavior was recorded for 10 min. The percentage of time spent and entrances into the central zone were scored, and the total distance travelled was determined using the ANY-maze video tracking program (Stoelting).

Y-maze test

The arena for Y-maze had three identical arms 30 cm in length and 5 cm in width with walls 20 cm high. The test animals were individually placed in the center, and the sequence of arm entries and total distance travelled over 5 min were recorded and analyzed by the ANY-maze video tracking program (Stoelting). The percentage of spontaneous alterations was calculated based on the number of triads containing entries into all three arms (ABC, ACB, BAC, BCA, CAB, CBA) divided by the maximum possible alterations (equivalent to the total number of arms entered minus 2) then multiplied by 100.

Tail suspension test (TST)

The mice were individually suspended by the tail in a box (36.5 x 30.5 x 30.5 cm) for 6 min. Recorded videos were analyzed by the ANY-maze video tracking program (Stoelting). Immobility was defined as the cessation of agitation and escape attempts by the mice.

Novel object recognition (NOR) test

The test arena was w40 x h40 x d40 cm. T-75 flasks filled with sand and stacked plastic bricks (w7 x l3 x h15 cm) were used as objects. The mice were individually habituated in the test arena without the objects for 10 min. On the next day, during acquisition, two identical objects were placed in the arena, and the individual mouse was allowed to explore freely for 10 min. The criterion for minimal exploration time for both identical objects during the acquisition phase was 20 s. The test phase was scheduled either 6 h (for the short-term memory test) or 24 h (for the long-term memory test) after acquisition. During the test phase, both a previously introduced object and a novel object were placed in the arena. The mice then were allowed to explore the arena for 10 min. The acquisition and test phases were recorded for analysis. The time spent exploring each object was measured. Exploration behavior was defined as showing interest towards the objects by sniffing.

Pavlovian fear conditioning

The mice were habituated for 10 min in a conditioning chamber (18 x 18 x 30 cm) the day before the acquisition phase. During acquisition, the mice were placed in the conditioning chamber and underwent 5 conditioning trial repetitions, each consisting of a tone (30 s, 5 kHz, 75 dB) that terminated with foot shocks (0.7 mA, 2 s) with an inter-trial interval of 60 s. After 24 h, conditioned fear responses were tested. For the contextual test, the conditioned mice were placed in the same

chamber without foot shocks and tones, and the freezing time was measured for 5 min. For the auditory test, the mice were placed in a distinct context and re-exposed to three tones at 90-s intervals without a foot shock after a 5-min period of exploration. Freezing behaviors were defined as immobility and scored during the tone presentation. The total freezing time in the test period was represented as a percentage of the average freezing duration for each tone presentation.

Unconditioned innate fear response test

The test animals were placed in a chamber (w18 x l18 x h30 cm) containing 30 μl synthetic predator fox feces odor (TMT, 2,5-dihydro-2,4,5-trimethylthiazoline, SRQ Bio, Florida, United States). After TMT exposure, TMT-evoked freezing behavior was recorded for 15 min and analyzed as the average percentage of freezing time at 3-min intervals using the ANY-maze video tracking program (Stoelting).

Supplementary Figures

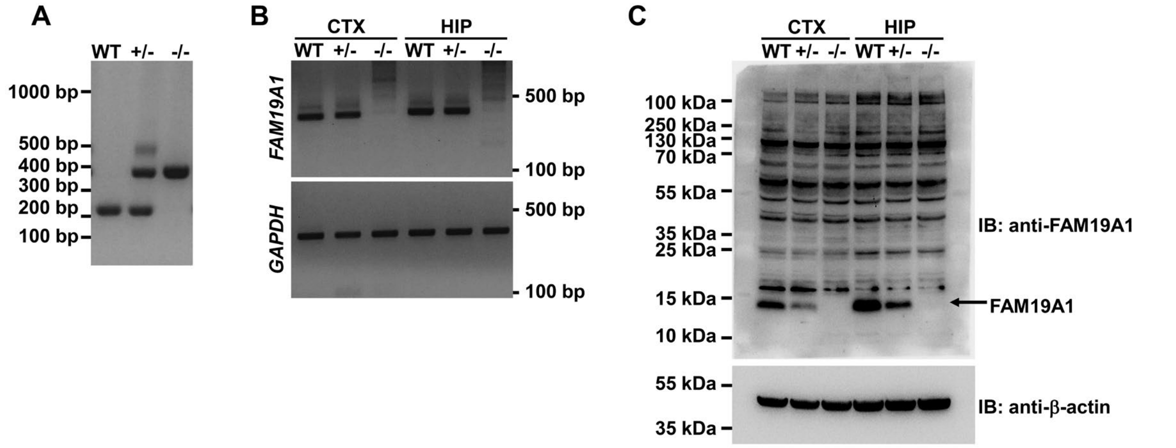

Supplementary Figure S1. Full-length gels and blots. (A) represents Fig. 1B. (B) represents Fig. 1C. (C) represents Fig. 1D. WT, wild type mouse; +/-, heterozygous *FAM19A1 LacZ* KI; -/-, homozygous *FAM19A1 LacZ* KI; CTX, cortex; HIP, hippocampus.

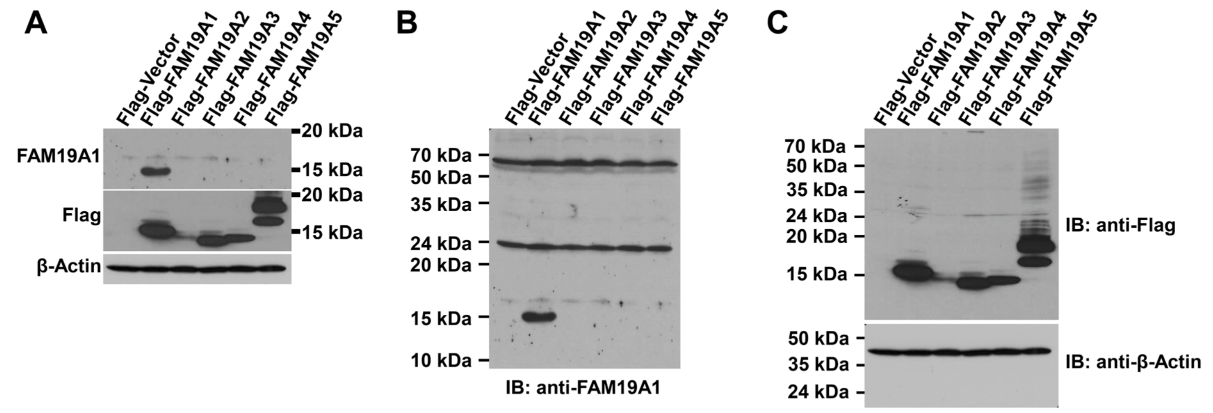

Supplementary Figure S2. Validation of FAM19A1 specific antibodies. (A) In-house generated FAM19A1 antibody was validated with Flag tagged FAM19A family overexpressed cell lysates. (B and C) Full-length blots for panel A. Exposure time during development with ECL solution; 5 min for FAM19A1, 15 min for Flag and 1 min for β-actin.

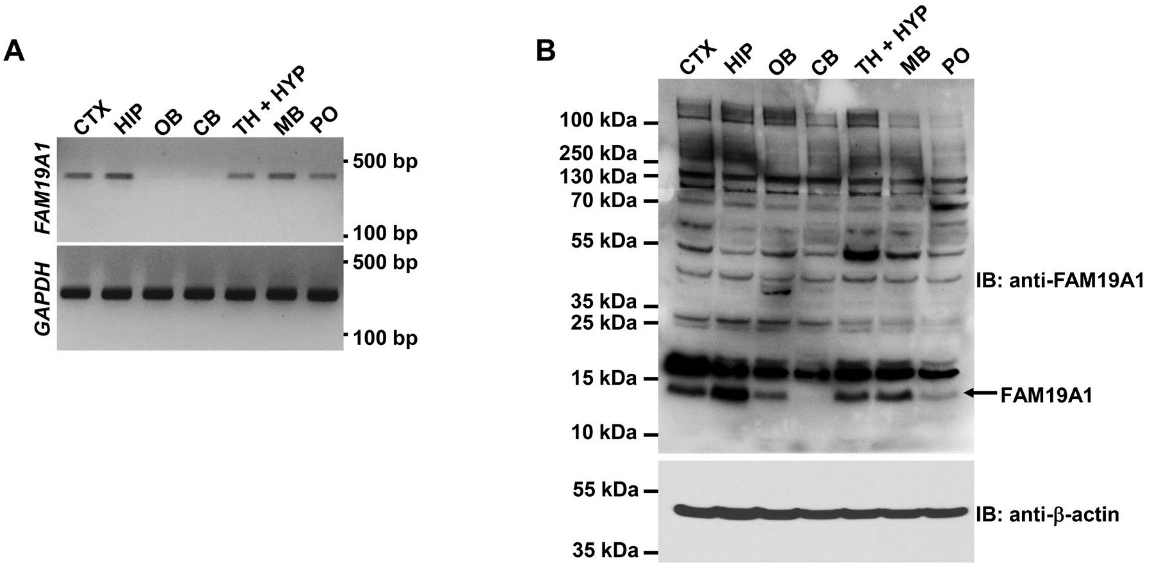

Supplementary Figure S3. Full-length gels and blots. (A and B) represent Fig. 1G. CTX, cortex; HIP, hippocampus; OB, olfactory bulb; CB, cerebellum; TH, thalamus; HYP, hypothalamus; MB, midbrain; PO, pons.

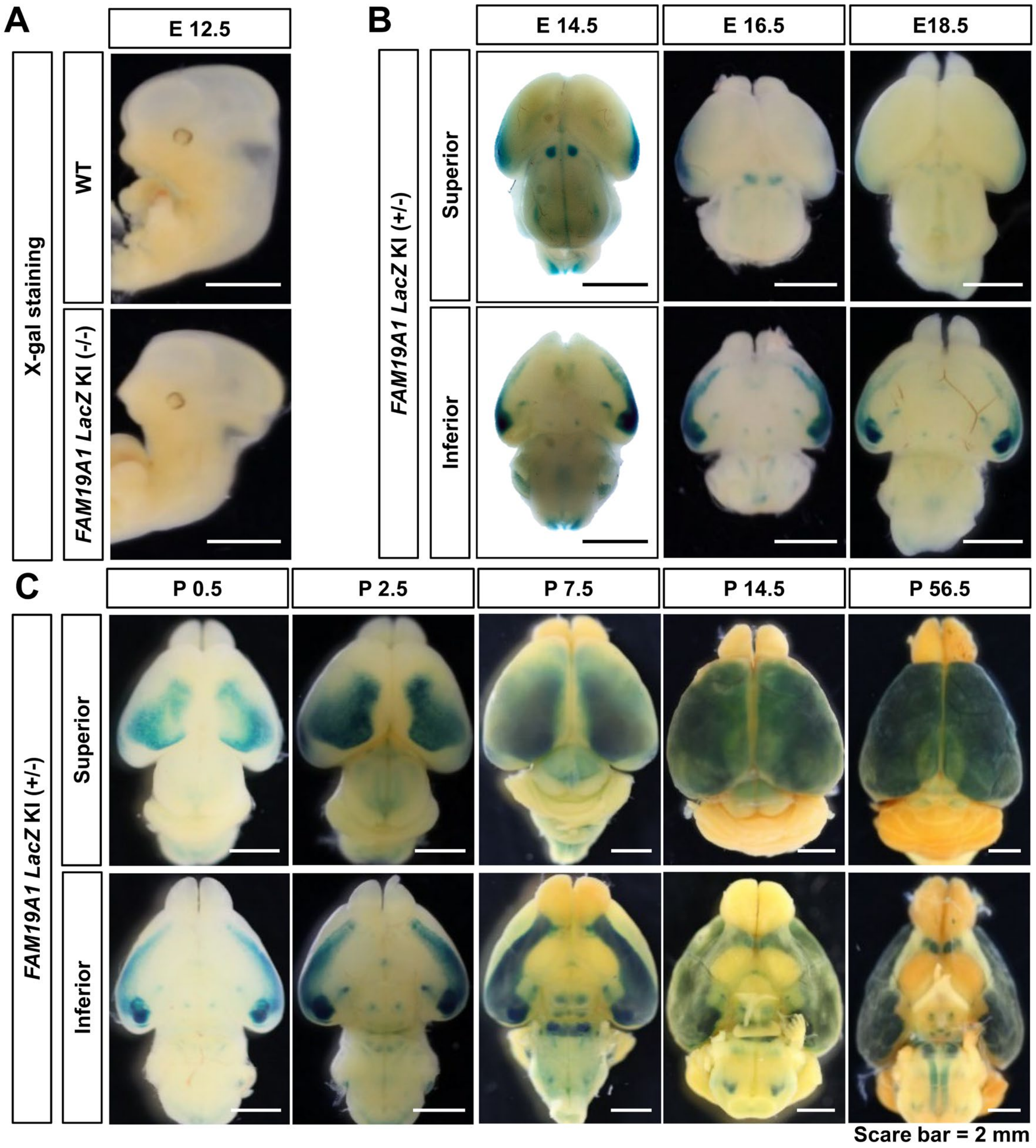

Supplementary Figure S4. Whole-brain X-gal staining of *FAM19A1 LacZ* knock-in (KI) mice across neurodevelopment. (A) Whole-mount X-gal staining of embryonic day 12.5 (E12.5) brains. (B) The expression of β-galactosidase during the embryonic stages starting from embryonic day 14.5 (E14.5). (C) The expression of β-galactosidase during the postnatal stages. Scale bars represent 2 mm.

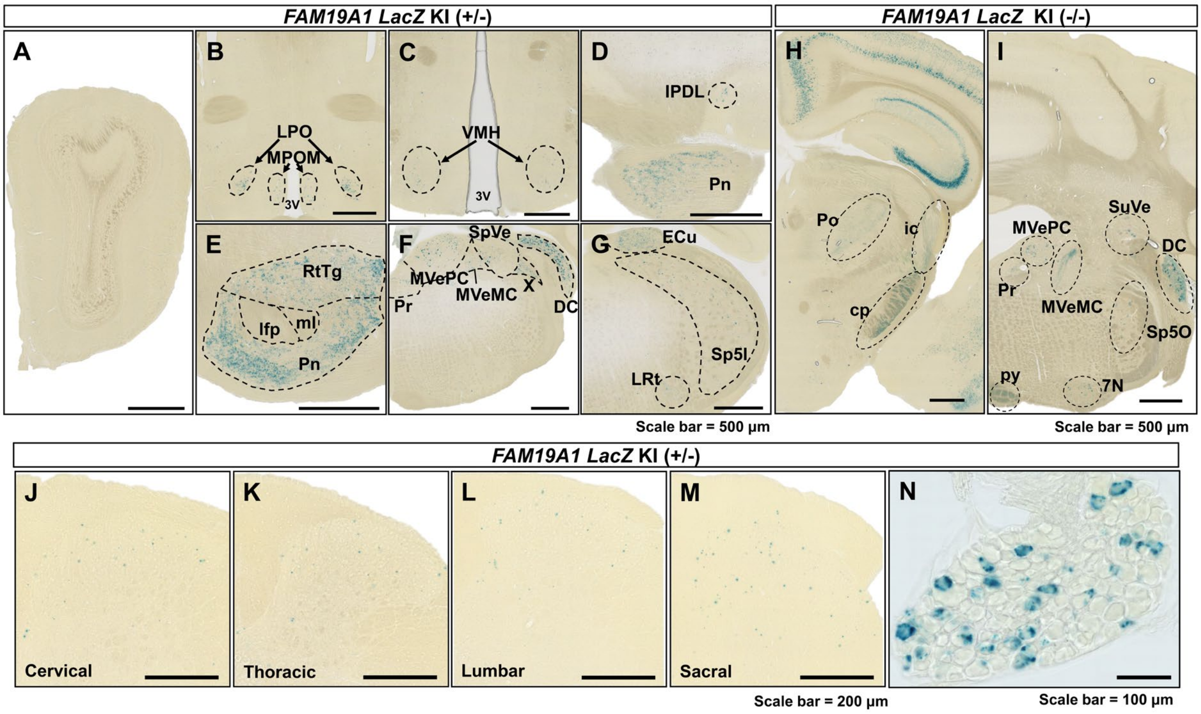

Supplementary Figure S5. *FAM19A1* expression in the adult mouse brain, spinal cord and dorsal root ganglia. (A~G) X-gal stained coronal brain sections of the heterozygous *FAM19A1 LacZ* knock-in (KI) mouse. (H and I) X-gal stained coronal brain sections of the homozygous *FAM19A1 LacZ* KI mouse. (J~M) X-gal stained coronal spinal cord sections of the heterozygous *FAM19A1 LacZ* KI mouse. (N) X-gal stained dorsal root ganglia of the heterozygous *FAM19A1 LacZ* KI mouse. 3V, 3rd ventricle; 7N, facial nucleus; cp, cerebral peduncle; DC, dorsal cochlear nucleus; Ecu, external cuneate nucleus; ic, internal capsule; IPDL, interpeduncular nucleus, dorsolateral subnucleus; lfp, longitudinal fasciculus of the pons; LPO, lateral preoptic area; LRt, lateral reticular nucleus; ml, medial lemniscus; MPOM, medial preoptic nucleus, medial part; MVeMC, medial vestibular nucleus, magnocellular part; MVePC, medial vestibular nucleus, parvicellular part; Pn, pontine nuclei; Po, posterior thalamic nuclear group; Pr, prepositus nucleus; py, pyramidal tract; RtTg, reticulotegmental nucleus of the pons; Sp5I, spinal trigeminal nucleus, interpolar part; Sp5O, spinal trigeminal nucleus, oral part; SpVe, spinal vestibular nucleus; SuVe, superior vestibular nucleus; VMH, ventromedial hypothalamic nucleus; X, nucleus X. Scale bars represent 500 μm in panels A to I, 200 μm in panels J to M and 100 μm in panel N.

A

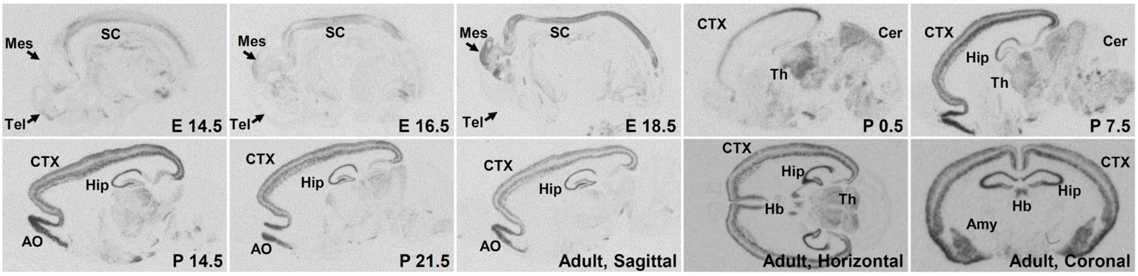

B

	WT	*FAM19A1* +/-	*FAM19A1* -/-	Total	Sex_ratio
Male	85 (26.9%)	153 (48.4%)	78 (24.7%)	316	0.51
Female	78 (25.2%)	158 (50.9%)	74 (23.9%)	310	0.49

Supplementary Figure S6. *FAM19A1* mRNA expression in the developing and mature wild type (WT) rat brains and the proportion of WT and *FAM19A1 LacZ* knock-in (KI) mouse genotypes. (A) *In situ* hybridization of the embryonic and postnatal WT rat brains with *FAM19A1* mRNA probes. (B) Numbers and percentages of offspring generated from the heterozygous *FAM19A1 LacZ* KI parents. Amy, amygdala; AO, anterior olfactory nucleus; Cer, cerebellum; CTX, cortex; Hb, habenular; Hip, hippocampus; Mes, mesencephalon; SC, spinal cord; Tel, telencephalon; Th, thalamus.

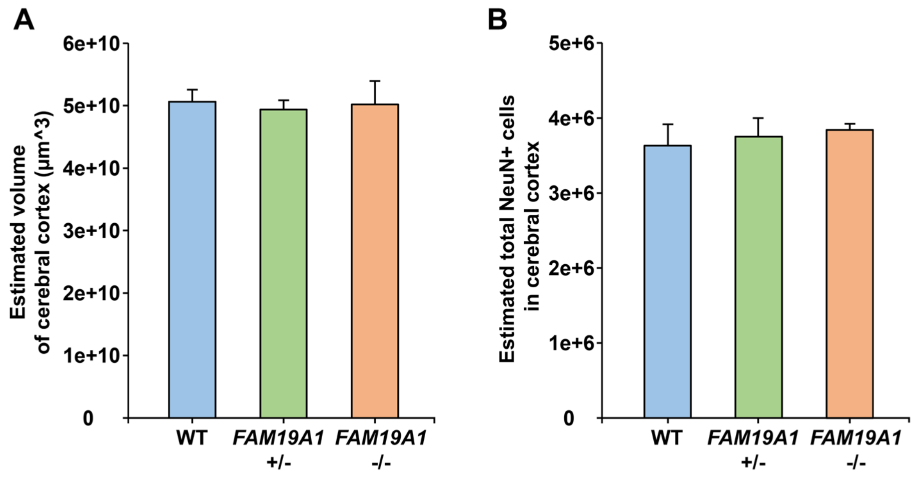

Supplementary Figure S7. Estimated cortical volume and total neuronal cell counts in the cerebral cortex of *FAM19A1 LacZ* knock-in (KI) adult mice. (A) Estimated volume of the cerebral cortex. (B) Estimated total NeuN-positive cells in the cerebral cortex. For all counts, WT (n =5), *FAM19A1* +/- (n = 5) and *FAM19A1* -/- (n = 5) mice were used. Data are presented as means ± standard errors of means (SEM).

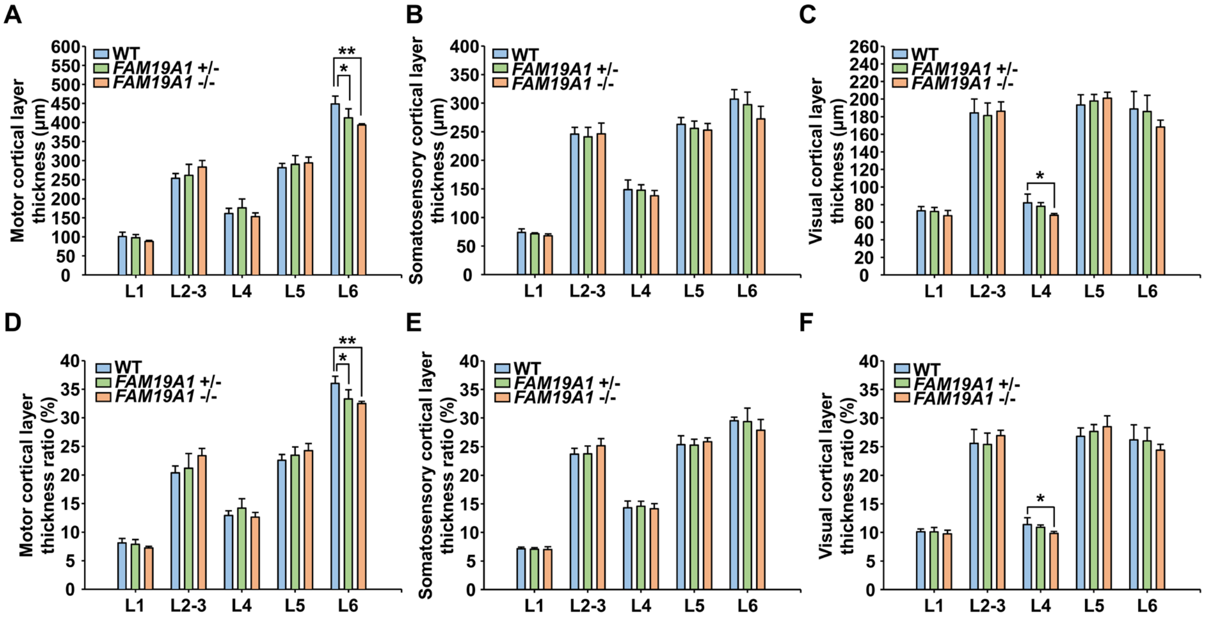

Supplementary Figure S8. Cortical layer thickness in *FAM19A1 LacZ* knock-in (KI) adult mice. (A, B and C) Cortical layer thickness of motor ($F(2, 11) = 10.00$, $p = 0.0034$), somatosensory and visual cortex ($F(2, 11) = 5.415$, $p = 0.0231$), respectively. (D, E and F) The thickness ratio of each cortical layer versus the total cortical thickness in the motor ($F(2, 11) = 10.06$, $p = 0.0033$), somatosensory and visual cortices ($F(2, 11) = 4.522$, $p = 0.0369$), respectively. For all counts, WT (n =5), *FAM19A1* +/- (n = 5) and *FAM19A1* -/- (n = 4) mice were used. Data are presented as means ± standard errors of means (SEM). *$p < 0.05$, **$p < 0.01$, ***$p < 0.001$ versus WT mice by one-way analysis of variance (ANOVA).

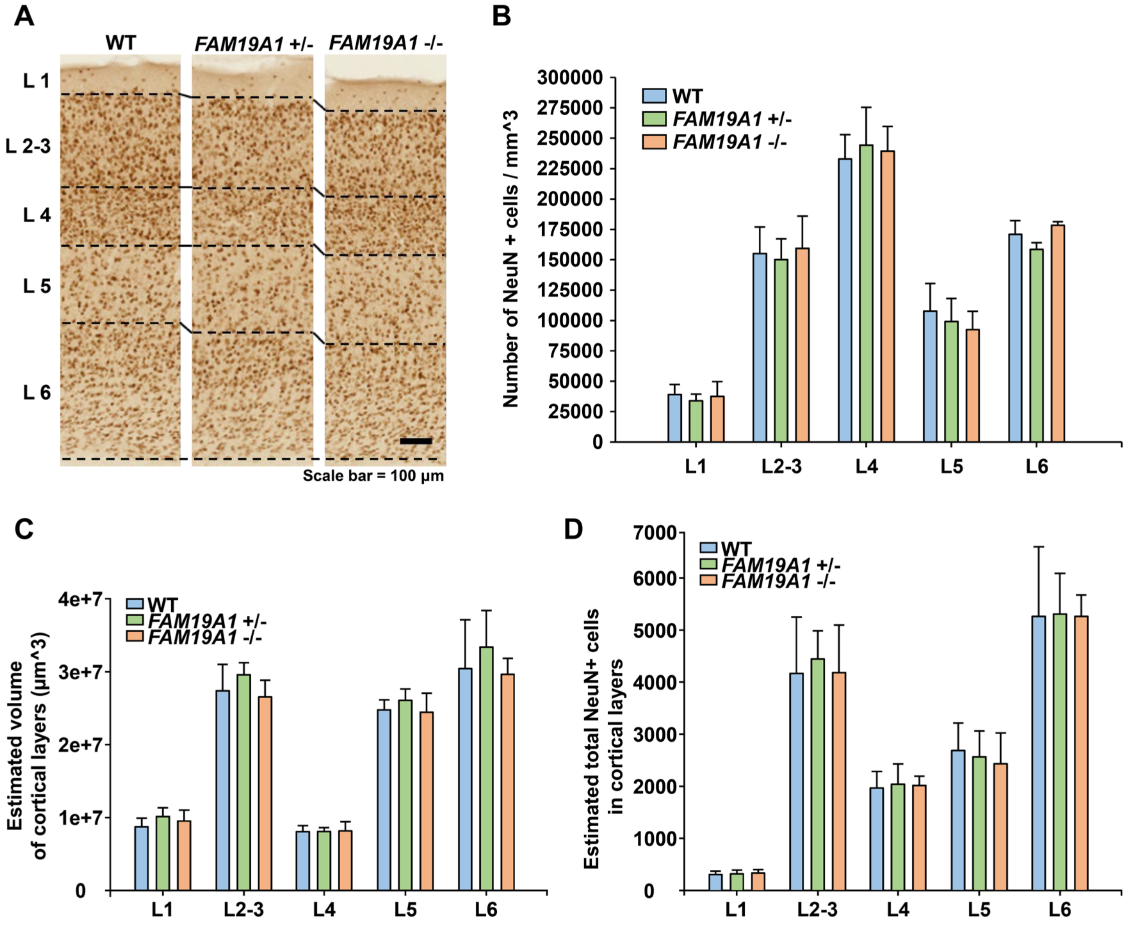

Supplementary Figure S9. Neuronal cell density in the motor cortical layers of *FAM19A1 LacZ* knock-in (KI) adult mice. (A) NeuN was used as a marker for neuronal cells. (B) Neuronal cell densities in each cortical layer were estimated. (C) Volumes of cortical layers were estimated. (D) Total NeuN-positive cells were estimated in each layer. For all counts, WT (n =4), *FAM19A1* +/- (n = 4) and *FAM19A1* -/- (n = 4) mice were used. Data are presented as means ± standard errors of means (SEM). Scale bars represent 100 μm in panel A.

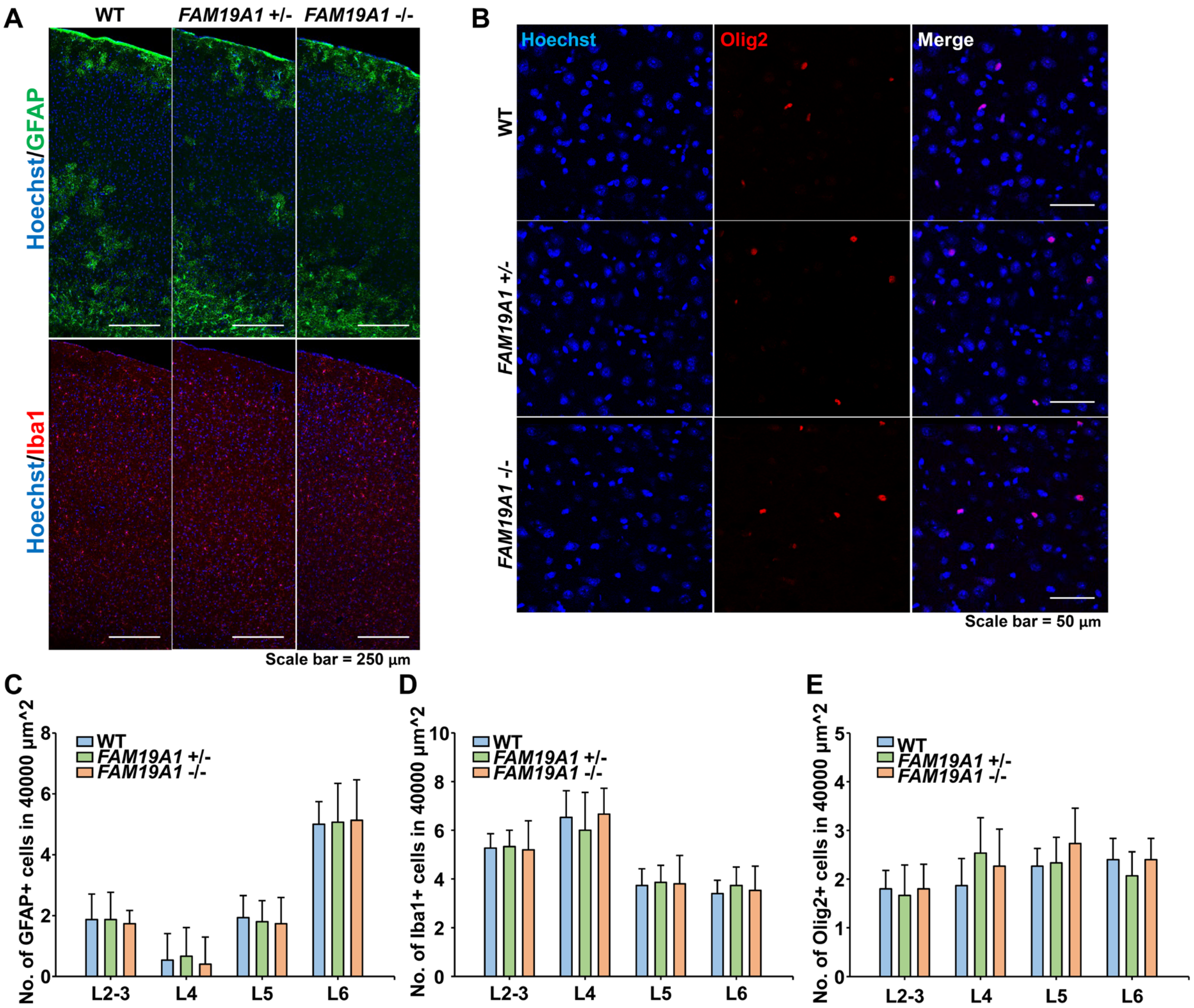

Supplementary Figure S10. Number of cortical layer glial cells in the motor cortex of *FAM19A1 LacZ* knock-in (KI) adult mice. (A) GFAP (green) positive astrocytes and Iba1 (red) positive microglia were detected in the motor cortex. (B) Olig2 (red) positive oligodendrocytes were identified in the motor cortex. (C) The number of GFAP-positive cells in the motor cortical layers. (D) The number of Iba1-positive cells in the motor cortical layers. (E) The number of Olig2-positive cells in the motor cortical layers. For all counts, WT (n = 5), *FAM19A1* +/- (n = 5) and *FAM19A1* -/- (n = 5) mice were used. Data are presented as means ± standard errors of means (SEM). Scale bars represent 250 µm in panel A and 50 µm in panel B.
